# Mosquitocidal and Oviposition Repellent Activities of the Extracts of Seaweed *Bryopsis pennata* on *Aedes aegypti* and *Aedes albopictus*

**DOI:** 10.3390/molecules200814082

**Published:** 2015-08-04

**Authors:** Ke-Xin Yu, Ching-Lee Wong, Rohani Ahmad, Ibrahim Jantan

**Affiliations:** 1 Drug and Herbal Research Centre, Faculty of Pharmacy, Universiti Kebangsaan Malaysia, 50300 Kuala Lumpur, Malaysia; E-Mail: yukxxin@gmail.com; 2 School of Biosciences, Taylor’s University, Taylor’s Lakeside Campus, Subang Jaya, 47500 Selangor, Malaysia; E-Mail: chinglee.wong@taylors.edu.my; 3 Medical Entomology Unit, Infectious Disease Research Centre, Institute for Medical Research, 50588 Kuala Lumpur, Malaysia; E-Mail: rohania@imr.gov.my

**Keywords:** dengue vector, ovicidal, larvicidal, insecticide, bis-(3-oxoundecyl) tetrasulfide

## Abstract

The ever-increasing threat from infectious diseases and the development of insecticide resistance in mosquito populations drive the global search for new natural insecticides. The aims of this study were to evaluate the mosquitocidal activity of the extracts of seaweed *Bryopsis pennata* against dengue vectors *Aedes aegypti* and *Aedes albopictus*, and determine the seaweed’s toxic effect on brine shrimp nauplii (as a non-target organism). In addition, the chemical compositions of the active larvicidal extract and fraction were analyzed by using liquid chromatography-mass spectrometry (LC-MS). Chloroform extract exhibited strong ovicidal activity (with LC_50_ values of 229.3 and 250.5 µg/mL) and larvicidal activity against *Ae. aegypti* and *Ae. albopictus*. The larvicidal potential of chloroform extract was further ascertained when its A7 fraction exhibited strong toxic effect against *Ae. aegypti* (LC_50_ = 4.7 µg/mL) and *Ae. albopictus* (LC_50_ = 5.3 µg/mL). LC-MS analysis of the chloroform extract gave a tentative identification of 13 compounds; Bis-(3-oxoundecyl) tetrasulfide was identified as the major compound in A7 fraction. Methanol extract showed strong repellent effect against female oviposition, along with weak adulticidal activity against mosquito and weak toxicity against brine shrimp nauplii. The mosquitocidal results of *B. pennata* suggest further investigation for the development of effective insecticide.

## 1. Introduction

Dengue fever and dengue hemorrhagic fever cause 50 to 100 million infection cases, with about 2.5% of those affected dying every year globally. Prior to 1970, only nine countries reported dengue cases, but presently at least 100 countries across Asia, Africa, America, Pacific and Caribbean islands claim to have endemic dengue [1]. Furthermore, earlier studies indicated that epidemic dengue occurs periodically every three to five years and most likely an increase in the magnitude and severity of cases with each new epidemic [2]. Besides the increasing number of infection cases and deaths, dengue fever also poses a growing burden to the economics of endemic and newly affected countries. For example, dengue infection in the Americas was estimated to cost US $2.1 billion per year [3]. Furthermore, dengue hemorrhagic fever is listed among the 10 leading causes of hospitalization in at least eight Asian countries [4]. Dengue fever and dengue hemorrhagic fever are mainly transmitted by mosquitoes, namely *Aedes aegypti* and *Aedes albopictus* in tropical countries. As female adults of *Ae. aegypti* and *Ae. albopictus* are anthropophilic, humans are at high risk of being targeted as hosts by the blood meal-seeking insect. *Aedes* mosquitoes become infected with dengue virus through feeding on infected humans and once the mosquitoes are infected, they remain infected for life. Furthermore, dengue virus also remains in the mosquito population by transovarial transmission [5].

There has been a heavy dependence on synthetic chemical insecticides for mosquito control programs since the discovery of chemical insecticides. The wide acceptance of chemical insecticides is due to their rapid effectiveness and convenience. However, prolonged usage of synthetic chemicals leads to undesirable effects, such as development of insecticide resistance in the vector population, environmental pollution and accidental poisoning of humans and non-target organisms [6]. Consequently, the search for alternative approaches in mosquito control has become crucial.

*Bryopsis pennata*, a seaweed species under the family of Bryopsidaceae, is widely distributed in tropical and temperate marine waters. *B. pennata* has green thallus with irregularly branched main axis, and forms tufts on rocks in the intertidal habitat or coral reefs [7]. This green seaweed has been found to exhibit antimicrobial activity towards pathogenic bacteria, fungi [8] and marine protists [9]. In addition, *B. pennata* also induces inotropic effect towards ventricular muscle strips of toad and positive chronotropic action towards isolated right atria of rat [10]. On top of that, the bioactive constituents of *Bryopsis* species have also been studied in various assays. For example, Kahalalides F, a polypeptide isolated from sacoglossan mollusk *Elysia rufescens* and its diet-green seaweed *Bryopsis* species [11], has been introduced into clinical phase trials as an anticancer agent against prostate cancer [12]. Biju *et al.* [13] reported that *Bryopsis plumosa* had antifeedant properties against larvae of moth *Hyblaea puera* and was able to reduce the protein and fat content of the treated larvae. 

Apart from having unique bioactive secondary metabolites with medicinal properties [14], seaweeds have been reported to have mosquitocidal properties [15,16,17]. Recently, the larvicidal activity of extracts and compounds of 30 seaweed species was described in the review of Yu *et al.* [18]. The few evaluations of mosquitocidal activities of seaweeds published provide limited information on the bio-efficacy of seaweed based insecticide, as compared to the usage of seaweeds in food and pharmaceutical applications. The findings of mosquitocidal properties of *B. pennata* are useful to the researchers of drug, insecticide and mosquito control programmes, as well as national governments and policy makers to prioritize their efforts. To help address this need, the present study evaluated the mosquitocidal potency of *B. pennata* against two dengue vectors, *Ae. aegypti* and *Ae. Albopictus*, and the toxic effect of this seaweed against non-target organism-nauplii of the brine shrimp *Artemia salina*, and characterized the seaweed by using liquid chromatography-mass spectrometry (LC-MS).

## 2. Results and Discussion

### 2.1. Seaweed Extraction

The dried seaweed yielded 17.16% ± 1.60% (*w*/*w*) of methanol extract. Liquid-liquid partition of methanol extract (12.0 g) gave to 5.8 g of hexane extract, 3.4 g of chloroform extract and 1.4 g of aqueous extract. VLC of chloroform extract yielded 8 fractions, namely A1 (0.1 g), A2 (0.6 g), A3 (0.3 g), A4 (0.2 g), A5 (0.5 g), A6 (0.4 g), A7 (0.3 g), and A8 (0.6 g). 

### 2.2. Mosquito Ovicidal Assay

The ovicidal assay of the dengue vectors *Ae. aegypti* and *Ae. albopictus* at 24 h post-treatment was tested with different concentrations of *B. pennata* extract, and the results are listed in Table 1. The chloroform extract of *B. pennata* was found to be the strongest ovicidal agent against *Ae. aegypti* and *Ae. albopictus* (exhibiting approximately 0.3 to 3.1-fold stronger activity than other extracts).

**Table 1:** Ovicidal activity of *Bryopsis pennata* extracts against *Aedes aegypti* and *Aedes albopictus*.

Mosquito	Extract	LC_50_ (µg/mL) (95% CL)	Slope (±SE)	*X*^2^
*Aedes aegypti*	*n*-Hexane	624.90 (579.10–674.40)	1.05 (0.03)	0.99
	Chloroform *	229.30 (167.50–313.90)	2.53 (0.61)	0.93
	Methanol	315.30 (252.30–394.20)	1.93 (0.35)	0.94
	Aqueous	939.80 (747.70–1181.00)	1.47 (0.11)	0.99
	Abate^®^ 1.1G	0.02 (0.01–0.03)	2.24 (0.47)	0.98
*Aedes albopictus*	*n*-Hexane	542.20 (460.30–638.80)	1.85 (0.20)	0.98
	Chloroform *	250.50 (190.10–330.20)	2.85 (0.69)	0.94
	Methanol	396.60 (319.80–491.90)	1.76 (0.30)	0.95
	Aqueous	691.60 (440.20–1087.00)	1.14 (0.20)	0.70
	Abate^®^ 1.1G	0.45 (0.21–0.63)	1.72 (0.26)	0.78

LC_50_, lethal concentration that kills 50% of the exposed eggs; 95% CL, 95% confidence limits; *X*^2^, chi-square value; * Extract with the strongest ovicidal effect.

### 2.3. Mosquito Larvicidal Assay

The larvicidal activity of *B. pennata* towards larvae of *Ae. aegypti* and *Ae. albopictus* at 24 h post-treatment was studied. The data clearly revealed that only chloroform extract had LC_50_ values below the concentration of 100 µg/mL (Table 2).

**Table 2:** Larvicidal activity of *Bryopsis pennata* extracts against *Aedes aegypti* and *Aedes albopictus*.

Mosquito	Extract	LC_50_ (µg/mL) (95% CL)	Slope (±SE)	*X*^2^
*Aedes aegypti*	*n*-Hexane	912.86 (821.04–1095.18)	5.25 (0.85)	1.12
	Chloroform *	92.72 (82.40–102.85)	3.01 (0.31)	3.14
	Methanol	156.97 (133.54–179.46)	2.57 (0.25)	1.47
	Aqueous	591.77 (528.19–692.70)	3.78 (0.49)	0.21
	Abate^®^ 1.1G	0.07 (0.06–0.08)	3.21 (0.21)	0.90
*Aedes albopictus*	*n*-Hexane	1209.50 (1123.50–1318.65)	3.15 (0.68)	1.83
	Chloroform *	99.85 (88.68–111.27)	2.81 (0.31)	0.65
	Methanol	177.50 (156.41–198.68)	3.05 (0.27)	4.02
	Aqueous	692.45 (657.01–735.71)	7.39 (0.84)	0.70
	Abate^®^ 1.1G	0.93 (0.82–1.07)	2.85 (0.34)	1.72

LC_50_, lethal concentration that kills 50% of the exposed larvae; 95% CL, 95% confidence limits; *X*^2^, chi-square value; * Extract with the strongest larvicidal effect.

As the most active extract of *B. pennata* in larvicidal assay, the chloroform extract was fractioned into 8 fractions and these fractions were subjected to mosquito larvicidal assay. Out of them, A7 was the strongest larvicidal fraction with approximately 5 to 68-fold stronger activity than others (Table 3).

**Table 3:** Larvicidal activity of the fractions derived from chloroform extract of *Bryopsis pennata* against *Aedes aegypti* and *Aedes albopictus*.

Mosquito	Fraction	LC_50_ (µg/mL) (95% CL)	Slope (±SE)	*X*^2^
*Aedes aegypti*	A1	324.50 (308.55–356.20)	2.68 (0.75)	0.96
	A2	226.10 (201.50–251.35)	2.45 (0.60)	0.95
	A3	189.33 (160.30–210.30)	2.86 (0.47)	0.94
	A4	209.45 (192.34–225.19)	2.13 (0.93)	0.91
	A5	146.43 (123.59–167.98)	2.21 (0.35)	0.96
	A6	26.81 (16.53–43.47)	1.18 (0.48)	0.48
	A7 *	4.65 (3.08–7.01)	1.29 (0.55)	0.68
	A8	254.29 (271.50–226.30)	2.11 (0.67)	0.96
	Abate^®^ 1.1G	0.07 (0.06–0.08)	3.21 (0.21)	0.90
*Aedes albopictus*	A1	256 (239.15–281.50)	2.11 (0.78)	0.90
	A2	227.80 (201.45–256.87)	2.62 (0.82)	0.98
	A3	181.55 (165.39–201.45)	1.88 (0.62)	0.96
	A4	193.45 (213.90–178.32)	1.89 (0.70)	0.95
	A5	128.40 (108.75–152.50)	1.73 (0.68)	0.98
	A6	38.78 (33.89–44.37)	3.98 (0.75)	0.88
	A7 *	5.32 (3.95–7.16)	1.87 (0.62)	0.81
	A8	278.90 (291.15–256.21)	1.99 (0.74)	0.90
	Abate^®^ 1.1G	0.93 (0.82–1.07)	2.85 (0.34)	1.72

LC_50_, lethal concentration that kills 50% of the exposed larvae; 95% CL, 95% confidence limits; *X^2^*, chi-square value; * Fraction with the strongest larvicidal effect.

Larvae treated with chloroform extract of *B. pennata* were observed to exhibit abnormal behaviour. These mosquito larvae showed signs of unnatural restlessness, wriggling movement and frequent sinking followed by floating, after 1–5 h of treatment. Such behaviour persisted until the larvae became sluggish, paralyzed and eventually sank to the bottom of the container. Mortality of the larvae was found to be on the rise from 5–20 h. Similar observations were noted for all larvae treated with different extracts except for the time and duration of exhibiting the intoxicated symptoms. 

**Figure 1:**
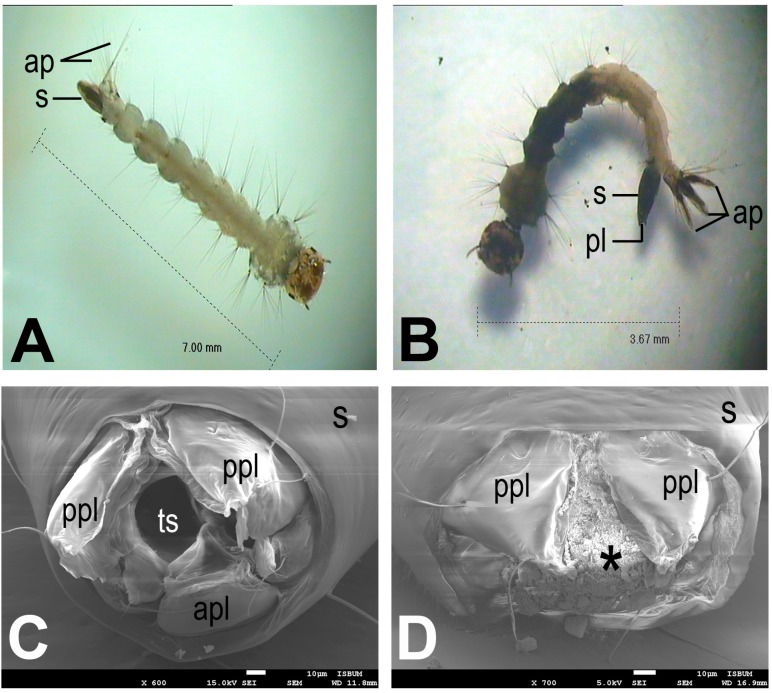
Photographs of *Aedes albopictus* larvae: (**A**) larva of negative control; (**B**) larva treated with chloroform extract of *Bryopsis pennata* showing darkened body parts and anal papillae; (**C**) larva of negative control showing intact spiracular apparatus; (**D**) larva treated with chloroform extract of *B. pennata* showing spiracular apparatus with damaged inner structures (*). ap, anal papillae; apl, anterior perispiracular lobe; pl, perispiracular lobes; ppl, posterior perispiracular lobe; s, siphon; ts, terminal spiracle.

In addition, larvae under the treatment of chloroform extract of *B. pennata* were observed to have darkened body segments and shrunken anal papillae as compared to the normal larvae. Further investigation under the electron microscope revealed that the treated larvae had spiracular apparatus with damaged inner structures (Figure 1). Similar observations were noted for all larvae treated with different extracts.

### 2.4. Mosquito Adulticidal Assay

The adulticidal activity of extracts of *B. pennata* against female mosquitoes at 24 h post-treatment is presented in Table 4. The chloroform extract was found to be the most effective adulticidal agent. No mortality was observed in negative control. The female adults treated with extract showed unusual restless movement and hardly remained still on the surface of the holding tube after being exposed to the treatment at high concentration. After 10–20 h of treatment, the female adults were increasingly found to wag, became paralyzed, lay at the bottom of the holding tube and then died.

**Table 4:** Adulticidal activity of *Bryopsis pennata* extracts against female adult of *Aedes aegypti* and *Aedes albopictus*.

Mosquito	Extract	LC_50_ (mg/cm^2^) (95% CL)	Slope (±SE)	*X*^2^
*Aedes aegypti*	Hexane	233.55 (200.34–294.34)	2.34 (0.90)	1.73
	Chloroform *	73.49 (64.89–85.34)	3.72 (0.81)	2.35
	Methanol	86.48 (76.76–97.42)	1.14 (0.05)	0.99
	Aqueous	523.82 (452.56–559.23)	1.34 (0.72)	1.45
	Malathion	0.01 (0.005–0.02)	1.785 (0.04)	0.96
*Aedes albopictus*	Hexane	434.32 (380.45–480.21)	3.45 (0.82)	1.93
	Chloroform *	100.32 (83.78–163.48)	1.39 (1.56)	1.34
	Methanol	156.34 (150.10–162.80)	1.41 (0.27)	0.99
	Aqueous	689.39 (602.34–723.43)	2.34 (2.36)	2.12
	Malathion	0.015 (0.009–0.021)	1.423 (0.03)	0.98

LC_50_, lethal concentration that kills 50% of the exposed adults; 95% CL, 95% confidence limits; *X^2^*, chi-square value; * Extract with the strongest adulticidal effect.

### 2.5. Mosquito Oviposition Assay

The oviposition activity of *B. pennata* against female adults of *Ae. aegypti* and *Ae. albopictus* (Table 5) proved its efficacy as oviposition repellent. The most effective repellency against mosquito gravid females was exhibited by methanol extract. Repellency of *B. pennata* increased with the increase of concentration. All extracts at five concentrations tested were observed to repel mosquitoes from oviposition, except for the aqueous extract at 50 and 100 µg/mL, hexane extract at 50 µg/mL and chloroform extract at 50 µg/mL, which exhibited no effect on the oviposition activity.

**Table 5:** Oviposition activity of *Bryopsis pennata* extracts against *Aedes aegypti* and *Aedes albopictus*.

Extract	Con. (µg/mL)	OAI (A/N/R) ^1^	Effective Repellency (ER)			
			Percentage (Mean ± SE) ^2^	Probit Analysis		
				RC_50_ (μg/mL) (95% CL)	Slope (±SE)	*X*^2^
*Aedes aegypti*						
Hexane	50	−0.28 (N)	42.40 ± 5.30 ^a^	60.78 (48.38–76.36)	1.73 (0.31)	0.83
	100	−0.53 (R)	69.54 ± 7.32 ^a^			
	200	−0.78 (R)	87.63 ± 8.32 ^a^			
	300	−0.90 (R)	94.98 ± 5.42 ^a^			
	400	−0.98 (R)	98.92 ± 4.76 ^a^			
Chloroform	50	−0.29(N)	46.55 ± 4.20 ^b^	54.58 (43.41–68.63)	1.85 (0.36)	0.82
	100	−0.59 (R)	74.17 ± 3.43 ^b^			
	200	−0.85 (R)	91.94 ± 9.60 ^b^			
	300	−0.93 (R)	96.23 ± 6.89 ^a,b^			
	400	−1.00 (R)	100.00 ± 5.23 ^a^			
Methanol *	50	−0.42 (R)	56.40 ± 6.51 ^c^	44.36 (35.21–55.85)	2.22 (0.55)	0.80
	100	−0.77 (R)	86.75 ± 7.91 ^c^			
	200	−0.90 (R)	94.62 ± 6.71 ^b^			
	300	−0.98 (R)	98.74 ± 2.58 ^b^			
	400	−1.00 (R)	100.00 ± 2.50 ^a^			
Aqueous	50	−0.19 (N)	29.86 ± 6.82 ^d^	106.30 (84.86–133.10)	1.58 (0.25)	0.85
	100	−0.26 (N)	41.06 ± 4.90 ^d^			
	200	−0.55 (R)	70.97 ± 9.37 ^c^			
	300	−0.76 (R)	86.19 ± 5.92 ^c^			
	400	−0.90 (R)	94.98 ± 2.34 ^b^			
*Aedes albopictus*						
Hexane	50	−0.20 (N)	32.49 ± 5.45 ^a^	70.67 (58.22–85.77)	1.79 (0.27)	0.87
	100	−0.54 (R)	70.50 ± 5.39 ^a^			
	200	−0.67 (R)	79.88 ± 7.31 ^a^			
	300	−0.88 (R)	93.44 ± 4.90 ^a^			
	400	−0.95 (R)	97.70 ± 2.33 ^a^			
Chloroform	50	−0.19 (N)	30.80 ± 5.10 ^a^	68.91 (58.06–81.80)	2.14 (0.35)	0.88
	100	−0.59 (R)	74.10 ± 3.43 ^a^			
	200	−0.76 (R)	86.39 ± 8.64 ^a,b^			
	300	−0.85 (R)	91.80 ± 3.51 ^a^			
	400	−0.97 (R)	98.62 ± 5.12 ^a^			
Methanol *	50	−0.29 (N)	47.50 ± 6.30 ^a^	51.60 (46.31–57.50)	2.81 (0.46)	0.91
	100	−0.78 (R)	87.77 ± 4.92 ^b^			
	200	−0.90 (R)	94.67 ± 2.36 ^b^			
	300	−0.98 (R)	98.91 ± 2.44 ^b^			
	400	−1.00 (R)	100.00 ± 2.56 ^a^			
Aqueous	50	−0.08 (N)	17.80 ± 4.58 ^a^	154.20 (129.70–183.50)	1.64 (0.21)	0.89
	100	−0.20 (N)	33.09 ± 2.90 ^c^			
	200	−0.37 (R)	53.85 ± 8.65 ^c^			
	300	−0.61 (R)	75.41 ± 3.74 ^c^			
	400	−0.81 (R)	89.40 ± 4.35 ^b^			

^1^ A, Attractant; N, No effect; R, Repellent; ^2^ Values followed by different letters within the same column of the same concentration are significantly different (*p* < 0.05); * Extract with the strongest oviposition repellent effect. RC_50_, concentration that causes 50% of the oviposition repellent activity; 95% CL, 95% confidence limits; *X^2^*, chi-square value.

### 2.6. Brine Shrimp Toxicity Assay

Table 6 shows that all *B. pennata* extracts exhibited very mild toxicity against the nauplii of *Artemia salina* (LC_50_ values above 500 µg/mL). Hexane extract of *B. pennata* was the most potent extract for the brine shrimp. It was observed that most nauplii became paralyzed and then died lying at the bottom of container, after 5–10 h of high-concentration treatment.

**Table 6:** Toxic effect of *Bryopsis pennata* extracts against nauplii of brine shrimp *Artemia salina*.

Extract	LC_50_ (μg/mL) (95% CL)	Slope (± SE)	*X*^2^
Methanol	1135.98 (901.50–1288.30)	2.98 (0.83)	0.99
Hexane *	591.82 (535.70–655.29)	0.22 (0.01)	0.98
Chloroform	911.50 (817.12–1055.17)	0.19 (0.01)	0.98
Aqueous	1238.23 (1198.50–1381.33)	2.12 (0.71)	0.91
Potassium dichromate	27.15 (25.07–29.41)	1.21 (0.06)	0.99

LC_50_, lethal concentration that kills 50% of the exposed nauplii; 95% CL, 95% confidence limits; *X^2^*, chi-square value; * Extract with the strongest lethal effect.

### 2.7. Liquid Chromatography-Mass Spectrometry Analysis

**Figure 2:**
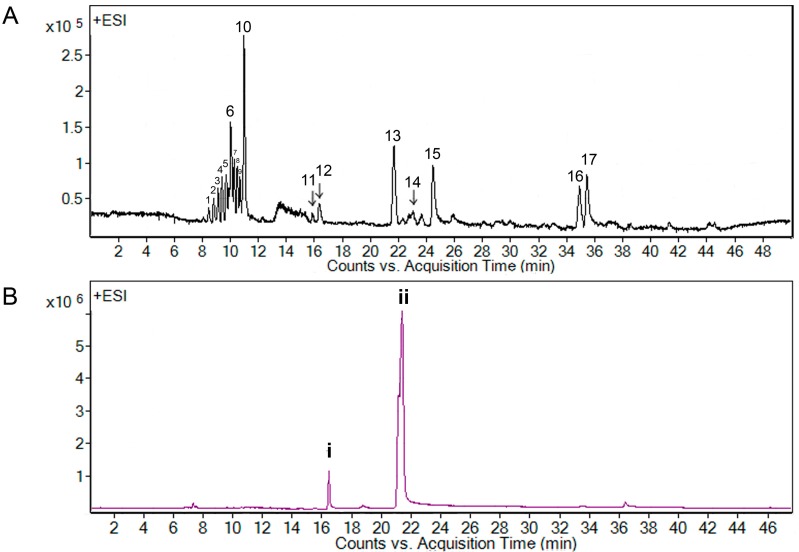
LC-MS extracted ion chromatogram of *Bryopsis pennata*, (**A**) chloroform extract and (**B**) A7 fraction.

Since chloroform extract and A7 fraction of *B. pennata* exhibited strong larvicidal activity, their chemical constituents were investigated and analyzed by using liquid chromatography-mass spectrometry (LC-MS). The LC-MS profile of the chloroform extract exhibited 17 peaks that were resolved in 36 min (Figure 2A), while the LC-MS profile of A7 fraction showed two main peaks (**i** and **ii**) (Figure 2B).

All the main peaks belonging to various compounds were tentatively assigned by comparing the data acquired by Mass Hunter Acquisition Data to Dictionary of Natural Products and Dictionary of Marine Natural Products (Table 7). Based on the comparison, peak **13** was tentatively assigned as an unbranched alkenic ketone, peaks **2** and **5** as alkaloids, peaks **3**, **6** and **11** as steroids, peak **4** as a meroterpenoid, peaks **9**, **10** and **17** as diterpenoids, peaks **14** and **16** as sesquiterpenoids, and peak **8** as a triterpenoid. The remaining four peaks did not match any data in the above mentioned dictionaries (peak **1**, **7**, **12** and **15**). Peak **i** (at 16.4 min) in A7 fraction and peak **12** (at 16.3 min) in the chloroform extract shared the same accurate mass at 768.4909; while peak **ii** (at 21.6 min) in A7 fraction corresponded to peak **13** (at 21.7 min) [assigned as bis-(3-oxoundecyl) tetrasulfide] in the chloroform extract, yielding an accurate mass of 466.3487. Peaks that shared similar retention times and accurate masses were suggested to be the same compound.

**Table 7:** LC-MS analysis and database search of main peaks in chloroform extract of *Bryopsis pennata* (Bryopsidales: Bryopsidaceae).

No	Rt (min)	Accurate Mass	Possible Molecular Formula	Possible Hits from Database	Type of Compound and Biological Source (Order: Family)
1	8.4	326.1923	C_19_H_34_O_4_	No hits	
2	8.8	370.2187	C_22_H_14_N_2_O_4_	Caulerpinic acid	Alkaloid of green seaweed *Caulerpa racemosa* (Bryopsidales: Caulerpaceae)
3	9.1	414.2442	C_29_H_50_O	Sitosterol	Steroid of green seaweed *Bryopsis plumose* (Bryopsidales: Bryopsidaceae)
4	9.4	340.2079	C_16_H_21_BrO_3_	Hydroxycymopochromenol	Meroterpenoid of green seaweed *Cymopolia barbata* (Dasycladales: Dasycladaceae)
5	9.7	384.2342	C_23_H_16_N_2_O_4_	Monomethyl caulerpinate	Alkaloid of green seaweed *Caulerpa racemosa* (Bryopsidales: Caulerpaceae)
6	10.0	428.2604	C_29_H_48_O_2_	Decortinol	Steroid of green seaweeds *Codium decorticatum* and *Codium arabicum*(Bryopsidales: Codiaceae)
7	10.2	472.2874	C_29_H_44_O_5_	No hits	
8	10.4	516.3135	C_34_H_60_O_3_	Botryolin A and B	Triterpenoid of microalga *Botryococcus braunii* (Trebouxiales: Botryococcaceae)
9	10.8	302.183	C_20_H_30_O_2_	2,6,10,14-Phytatetraene-1,20-dial	Diterpenoid of green seaweed *Caulerpa brownie* (Bryopsidales: Caulerpaceae)
10	11.1	346.2087	C_22_H_34_O_3_	2,6,10,14-Phytatetraene-1,20-diol, Variant: (2E,6E,10E)-form, Derivative: 1-Aldehyde, 20-Ac	Diterpenoid of green seaweed *Caulerpa brownie* (Bryopsidales: Caulerpaceae)
11	15.8	444.2243	C_29_H_48_O_3_	7-Hydroperoxystigmasta-5,25-dien-3-ol	Steroid of green seaweed *Codium arabicum* (Bryopsidales: Codiaceae)
12	16.3	768.4909	C_43_H_60_O_12_	No hits	
13	21.7	466.3487	C_22_H_42_O_2_S_4_	Bis-(3-oxoundecyl) tetrasulfide *	Unbranched alkenic ketone of brown seaweed *Dictyopteris* spp.(Dictyotales: Dictyotaceae)
14	23.0	278.1494	C_17_H_26_O_3_	4-Hydroxy-2-[2-(2,6,6-trimethyl-2-cyclohexen-1-yl)ethyl]-2-buten-1-al, Derivative: Ac	Sesquiterpenoid of green seaweed *Caulerpa flexilis* (Bryopsidales: Caulerpaceae)
15	24.4	921.0025	C_54_H_83_NO_11_	No hits	
16	34.9	390.2742	C_21_H_26_O_7_	10,11-Epoxycaulerpenyne	Sesquiterpenoid of green seaweed *Caulerpa taxifolia* (Bryopsidales: Caulerpaceae)
17	35.4	390.2749	C_24_H_38_O_4_	Trifarin	Diterpenoid of green seaweed *Caulerpa flexilis* (Bryopsidales: Caulerpaceae)

* Suggested compound for peak **13** of chloroform extract LC-MS profile and peak **ii** of fraction A7 LC-MS profile.

### 2.8. Discussion

Development of new insecticides based on natural products requires a thorough understanding of the potential activity of natural products against mosquito at each stage of the insect’s life. Furthermore, the information on toxic effect of bioinsecticides against non-target organisms serves a useful basis for the development of safer and more selective mosquitocidal agents. In this study, dengue vectors of *Ae. aegypti* and *Ae. albopictus* were used as a model system and brine shrimp nauplii was used as non-target organism, to investigate the potential of different extracts of seaweed *B. pennata*.

Applications of ovicide and larvicide are effective strategies to control the population of mosquito since controlling the egg and larva that live in bounded aquatic area is easier compared to targeting the free-flying adult [19]. Furthermore, as dengue virus is transmitted transovarially by mosquito, ovicide could be the solution to suppress the vector population. Therefore, inhibiting the egg hatchability, larval emergence, and oviposition of gravid female are the main aims of ovicidal agent in mosquito control. Previous studies showed that treatment of plant extract induces morphometric changes to the mosquito egg that inhibits the development of the egg, such as swelling with increase in length of the egg and deformities in air floats [20]. Furthermore, studies also revealed that plants had promising ovicidal and oviposition repellent effects against *Ae. aegypti* [21,22,23]. However, ovicidal and oviposition repellent potentials of seaweeds have not been studied much. Interestingly, the ovicidal and oviposition repellent properties of chloroform and methanol extracts of *B. pennata* in our report are comparable to that of other reports [21,24].

Earlier studies have suggested that seaweeds also exhibit skin repellent and smoke repellent properties against adult mosquitoes [25,26], but little information about the adulticidal properties of seaweed. In our report, chloroform extract of *B. pennata* resulted in the strongest adulticidal effect among the extracts tested, but it was considered as an ineffective adulticidal agent against *Ae. aegypti* when compared to extract of monocot flowering plant *Acorus calamus* (LC_50_ value of 0.04 mg/cm^2^) and essential oil of wild sage *Lantana camara* (LC_50_ value of 0.06 mg/cm^2^) [27,28]. On the other hand, the mosquitocidal activities of bioinsecticides are comprised of both toxic and behavioural effects [29]. In the present study, the observation of abnormal behaviour changes of adult females after treatment of seaweed extract is in agreement with previous studies using other plant extracts [28,30] and these symptoms were also similar to those caused by nerve poison [31].

The use of seaweeds as effective mosquito larvicide has been reported by researchers [18,32]. Furthermore, Bianco *et al.* [17] reported that hexane extract of red seaweed *Laurencia dendroidea* had strong larvicidal effect by causing 100% mortality at 50 ppm against *Ae. aegypti* larvae. Sequential fractionation of the hexane extract of *L. dendroidea* yielded elatol that exhibited LC_50_ value of 10.7 ppm against *Ae. aegypti* larvae. This demonstrates that pure compound has higher efficiency in larvicidal activity when separated from the extract (with a combination of compounds), due to a higher concentration of the compound being available for bioactivity action. Our findings are in line with the finding of Bianco *et al.* [17] showing A7 fraction of *B. pennata* exhibited more than 15-fold stronger larvicidal effect as compared to the extracts. In addition, the larvicidal activity of A7 fraction in the present study is comparable to other larvicidal compounds derived from either terrestrial plants or seaweeds [18,33,34].

Apart from that, β-sitosterol (the anomer of compound which assigned as peak **3** in the LC-MS analysis of chloroform extract), was reported as an active mosquito larvicidal compound isolated from the petroleum ether extract of shrub *Abutilon indicum* in a previous report with the LC_50_ value of 11.49 ppm against *Ae. aegypti* [35]. However, the presence of sitosterol in the present study did not have a significant effect on the larvicidal action as compared to the previous study. The bioactivity of plant extracts and fractions depends on the biomass production and chemical composition which highly related to the natural variability and sample preparation [18,36]. Therefore, it is suggested that sitosterol may have a lower concentration in the extracts of *B. pennata* due to different species and extraction methods; hence the weak larvicidal effect on mosquitos in the present study.

**Figure 3:**
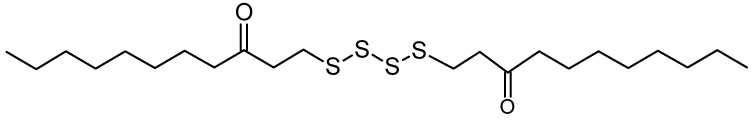
Bis-(3-oxoundecyl) tetrasulfide (possible hit of Peak **13** or **ii** from Dictionary of Marine Natural Products).

In view of the active larvicidal activity of the A7 fraction, the compounds present in the A7 fraction were assumed to correlate with the strong mosquito larvicidal effect. The match of peak **13** [assigned as bis-(3-oxoundecyl) tetrasulfide] or **ii** of LC-MS analysis in the dictionary suggested that the compound has an unbranched long hydrocarbon chain with carbonyl group (Figure 3). This is in line with the previous reports that described active mosquito larvicidal compounds with their chemical characteristics such as lipophilic profile [37] and possession of double bond [38]. For example, aliphatic fatty acids with a long hydrocarbon chain derived from green seaweed *Cladophora glomerata* (having LC_50_ values of 3‒14 ppm against *Aedes triseriatus*) [39] and alkaloids with double bond isolated from green seaweed *Caulerpa racemosa* (having LC_50_ values of 1.4‒4.8 ppm against *Culex pipiens*) [40]. Further confirmation of the identity of peak **13** or **ii** and its larvicidal activity is warranted, as the literature on bis-(3-oxoundecyl) tetrasulfide is limited [41,42].

In spite of exhibiting killing action towards mosquito at different stages of its life cycle, seaweed has been proven to have deleterious impact on the morphological structure and behaviour of the treated mosquitoes [17,32]. These symptoms were noted in our observation of the study. Structural alteration of anal papillae of mosquito larvae leads to its dysfunctionality which may result an interruption of osmosis and ionic regulations [43,44]. Osmosis and ionic imbalance of the mosquito larvae may be intrinsically associated with the larval death or may be part of the mechanism causing the death of mosquito larvae [45]. Furthermore, the rupture of larva’s inner structure of spiracular apparatus observed in the present report is suggested to cause destruction to the hydrophobic surface of stigmal plate, causing water/medium to enter the tracheal trunk which harms the respiration system of the larvae [46,47]. On the other hand, the abnormal behaviour of intoxicated larvae might be due to the effect of insecticidal extracts that affects the neuromuscular coordination in chemical synapses [48]. In addition, interaction with the picrotoxinin receptor of insect central nervous system (cyclodiene-type mechanism) is proposed to be one of the action modes of insecticidal compounds derived from seaweeds [49]. 

An effective mosquitocidal agent should be target-specific but pose little risk to the non-target organism. Therefore, the brine shrimp nauplii toxicity test offers a relatively rapid and convenient method to assess the toxic effect of natural products [50]. Various seaweeds have been tested for their toxicity against brine shrimp nauplii [18]. In our report, *B. pennata* induced strong larvicidal effect towards *Aedes* mosquito but weak toxic effect towards brine shrimp nauplii. A similar trend was observed in the study of brown seaweed *Padina gymnospora* reported by Guedes *et al.* [51].

To the best of our knowledge, this is the first report on the mosquitocidal properties of green seaweed *B. pennata* against dengue vectors *Ae. aegypti* and *Ae. albopictus*. Although the present study demonstrated the mosquitocidal potential of *B. pennata*, identification of active compounds and their mechanism of action, as well as their possible synergistic effect, may allow the development of bioinsecticides with greater potency than the extract and fraction evaluated here. Chemical synthesis of the active larvicidal analogues and formulation of binary insecticides may also provide useful end products. In addition, the insecticide should be tested on different mosquito species and in the field to ensure its efficacy. 

## 3. Experimental Section

### 3.1. Preparation of Seaweed Extracts

**Figure 4:**
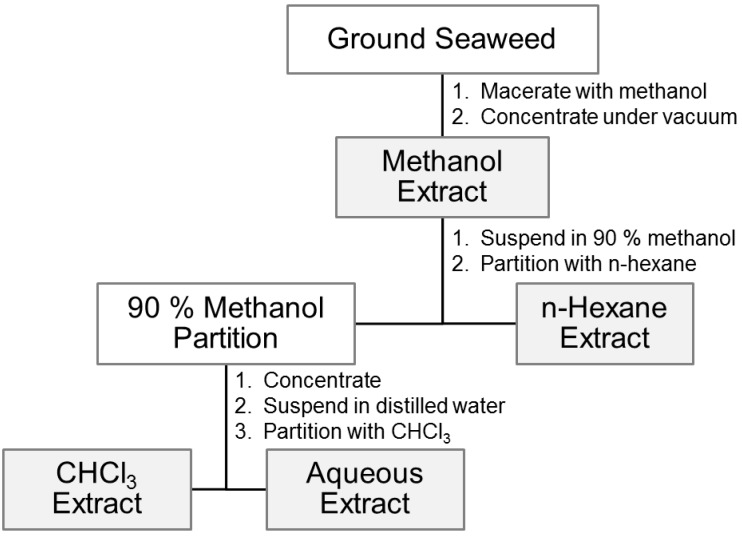
Procedure for preparing extracts representing a range of polarities. Source: Adapted from Jones and Kinghorn [53].

Fresh *B. pennata* (voucher number: CRM-C1) was collected in October to December of 2012 from Teluk Kemang (2°26.29′ N, 101°51.42′ E), Port Dickson, Malaysia. The sample was identified and voucher specimen was deposited at the herbarium maintained by Faculty of Science and Technology, Universiti Kebangsaan Malaysia. Dried sample was ground, sieved, macerated for 72 h with methanol (Merck, Darmstadt, Germany) (60 g/L), and stirred with the aid of magnetic stirrer. The sample was extracted 3 times. Then, the solvent was filtered and concentrated by using Rotavapor^®^ R-210 rotary evaporator (Buchi, Flawil, Switzerland) at 50 °C to dryness to yield the methanol extract [52]. The methanol extract was liquid–liquid partitioned to hexane, aqueous and chloroform extracts [53] (Figure 4). These extracts were again concentrated by using rotary evaporator and kept in vials at 4 °C prior to mosquito assay. The most active extract of *B. pennata* in mosquito larvicidal assay was fractioned by using vacuum liquid chromatography (VLC) (Rocker Scientific Co., Ltd., Taipei, Taiwan). Then, the fractions were combined based on the pattern of thin layer chromatography (TLC) (Merck).

### 3.2. Maintaining Mosquito Culture 

Laboratory strains of *Ae. aegypti* and *Ae. albopictus* were obtained from the insectary of the Institute for Medical Research (IMR), Malaysia. The colony was maintained at the temperature of 26 ± 1 °C and relative humidity of 80% ± 5%. The mosquito larvae were reared in a plastic tray filled with de-chlorinated water and fed with liver powder and half cooked beef liver. Pupae were collected and transferred into an enamel bowl filled with water which was later placed in a mosquito cage (30 × 30 × 30 cm) for adult emergence. Adults were provided with 10% sucrose solution mixed with Vitamin B, and restrained mice for blood meal. Bowls containing de-chlorinated water and fitted with filter paper were placed in the mosquito cage as ovitrap to collect egg. The procedures for colonization, feeding and use of mosquitoes of Entomology Unit, Infectious Disease Research Centre, IMR have been followed. These procedures were in accordance with the Section 14, Destruction of Disease-Bearing Insects Act 1975 (amended 2000) and approved by the Ministry of Health, Malaysia in 2006.

### 3.3. Mosquito Ovicidal Assay

Freshly laid intact mosquito eggs (30 to 50 eggs per batch) of *Ae. aegypti* and *Ae. albopictus* were exposed to seaweed extract solutions of different concentrations (100 to 500 µg/mL) in 50-mL container for 5 h. After that, the eggs were transferred into paper cups filled with distilled water and allowed for hatching [21]. The seaweed extract solutions were prepared in 0.1% (*v*/*v*) of methanol solution. Larvae that emerged from the treated eggs were counted under Leica EZ4HD stereomicroscope (Leica Microsystems Inc., Buffalo Grove, IL, USA) every 24 h for 7 days before being removed from the container. Eggs with unopened opercula were considered as unhatched/dead eggs. Ovicidal activity was calculated as percentage of unhatched/dead egg after 7 days. Negative control of the experiment was prepared by soaking the eggs from the same batch in 0.1% (*v*/*v*) of methanol solution, while positive control was prepared by soaking the eggs into Abate^®^ 1.1G (Temephos 1.1% *w*/*w*) (0.011 g/mL) (BASF (Malaysia) Sdn. Bhd., Shah Alam, Selangor, Malaysia). The experiment was carried out 3 times with triplicates. The LC_50_ value and chi-square were calculated by using the probit analysis of IBM SPSS Statistics version 20 software (IBM Corp., Armonk, NY, USA).

### 3.4. Mosquito Larvicidal Assay

The larvicidal assay was conducted according to the guidelines of the World Health Organization [54] with slight modifications. The larvicidal assay was performed by using extracts and followed by the fractions (yielded from the active extracts). The fourth instar larvae were divided into a few batches with each batch constituting 25 larvae. They were then put into different 200 mL-paper cups filled with extract/fraction of various concentrations, which had been prepared through dilution of stock solution in distilled water. Abate^®^ 1.1G (Temephos 1.1% *w*/*w*) (0.011 g/mL) (BASF (Malaysia) Sdn. Bhd.) was used as positive control and 0.1% (*v*/*v*) of methanol was used as negative control. The behaviour of the treated larvae was observed hourly for 24 h. The larvae were considered dead if they did not move when the water was disturbed. The larval mortality was recorded at the end of 24-h monitoring. The experiment was repeated 5 times with triplicates. The LC_50_ value and chi-square were calculated by using the probit analysis of IBM SPSS Statistics version 20 software (IBM Corp.).

The morphology of treated larvae was examined and recorded by using stereo microscope and scanning electron microscope (SEM) after the 24-h treatment. For stereo microscope study, the larvae were fixed in 80% ethanol and observed under stereomicroscope Leica EZ4HD (Leica Microsystems Inc., Buffalo Grove, IL, USA) [55]. SEM study was done according to Neves Filho *et al.* [47] with modifications. The larvae were rinsed with distilled water, followed by 8% of glutaraldehyde and Sorensen’s phosphate buffer (for 1 h), Sorensen’s phosphate buffer and distilled water (1:1) (for 1 h), and 4% of osmium tetroxide (Sigma-Aldrich, St. Louis, MO, USA) and distilled water (1:3) at the temperature of 4 °C (for 14 h). After that, the larvae were subjected to dehydration in serial alcohol and acetone, followed by drying by using critical point dryer CPD300 (Leica Microsystems Inc.), and subsequently the larvae were mounted. Then, the larvae were spurted with 45 nm gold for 1 min by using auto fine coater JFC-1600 (JEOL Ltd., Tokyo, Japan). Finally, the larvae were viewed and recorded by using thermal field emission scanning electron microscope JSM-7001F (JEOL Ltd.).

### 3.5. Mosquito Adulticidal Assay

Adulticidal assay was carried out according to the guidelines of World Health Organization [56] with slight modifications. Stock solutions (10,000 µg/mL) were prepared by dissolving seaweed extract in methanol solution. Impregnated papers were prepared freshly prior to testing. Each filter paper (140 × 115 mm) was impregnated with 4 mL of solution making final concentrations of 0.248, 0.496, 0.993 and 1.987 mg/cm^2^. Then, the impregnated papers were left to air-dry at temperature of 25 ± 2 °C. Malathion impregnated filter paper (WHO, Geneva, Switzerland) was used as a positive control at a diagnostic dosage of 5% (*v*/*v*). The filter papers used for negative control were impregnated with 5% (*v*/*v*) methanol solution. Different batches formed by 15 adult females each were introduced to the exposure tubes (WHO, Geneva, Switzerland) with an impregnated paper in each tube for 3 h. At the end of the 3-h exposure, the mosquitoes were transferred to a holding tube (WHO) and given 10% sugar solution enriched with Vitamin B complex as food. The behaviour of the treated adults was observed hourly. The mosquito was considered dead if it showed no response, no sign of movement and lying on the holding tube. Mortality was recorded after 24 h. The experiment was repeated 3 times with triplicates. The LC_50_ value and chi-square were calculated by using the probit analysis of IBM SPSS Statistics version 20 software (IBM Corp.).

### 3.6. Mosquito Oviposition Assay

Gravid female adults which have given blood meal 3 days ago were released into mosquito cages (30 × 30 × 30 cm) (15 females per cage). Solutions of seaweed extract were prepared in different concentrations (50–400 µg/mL). Plastic bowl containing filter paper folded in cone shape (as oviposition site) and 50 mL of test solution was used as ovitrap. One control ovitrap (containing 0.20% *v*/*v* of methanol) and one test ovitrap (containing seaweed extract) were placed diagonally at the opposite corners of each mosquito cage. The positions of ovitraps were rotated between the different replicates to counteract the position effect. Filter paper in the ovitrap was replaced every 24 h for 3 days and the number of eggs laid on the filter paper were counted under stereomicroscope Leica EZ4HD (Leica Microsystems Inc.). The experiment was carried out 3 times with triplicates [21]. All experiments were conducted at temperature of 26 ± 2 °C and relative humidity of 80% ± 2%. One-way analysis of variance (ANOVA) followed by Tukey test of IBM SPSS Statistics version 20 software (IBM Corp.) were used to determine significant differences between the treatments.

Effective repellency (ER%) [57] and oviposition active index (OAI) [58] were calculated by using the following formulas:

(1) $$\text{ER\%}=\frac{\left(\text{NT}-\text{NC}\right)}{\text{NC}}\times 100$$

(2) $$\text{OAI}=\;\frac{\left(\text{NT}-\text{NC}\right)}{\left(\text{NT}+\text{NC}\right)}$$

NT is the total number of eggs laid in the extract solution, and NC is the total number of eggs laid in the control solution.

The treatment with OAI value of +0.3 or above is considered as oviposition attractant while the treatment with OAI value of −0.3 or below is considered as oviposition repellent [58]. Positive value indicates that the test solution was an attractrant for oviposition, as more eggs were deposited in the test ovitrap than in the control ovitrap. On the other hand, negative value indicates that the test solution was a deterrent for oviposition, as more eggs were deposited in the control ovitrap than in the test ovitrap [21].

The RC_50_ value (concentration that caused 50% repellency) and chi-square were calculated by using the probit analysis of IBM SPSS Statistics version 20 software (IBM Corp.). One-way analysis of variance (ANOVA) followed by Tukey test using IBM SPSS Statistics version 20 software was used to determine significant differences between the treatments.

### 3.7. Brine Shrimp Toxicity Assay 

Each batch of 10 newly hatched *Artemia salina* nauplii was introduced to 5 mL of seaweed extract solution with concentrations ranging from 300 to 600 µg/mL. The seaweed extract solution was prepared by using stock solution (20 mg/mL) and brine medium (15 mg of sea salt in 1 mL of distilled water). Three replicates were prepared for each test sample and the experiment was repeated 3 times [50]. Potassium dichromate solution (25 μg/mL) (Sigma-Aldrich.) was used as a positive control and 0.1% (*v*/*v*) methanol in brine medium was prepared as negative control. The mortality of nauplii was recorded after 24 h. The LC_50_ value and chi-square were calculated by using the probit analysis of IBM SPSS Statistics version 20 software (IBM Corp.).

### 3.8. Liquid Chromatography-Mass Spectrometry Analysis

The sample was prepared in methanol with the concentration of 20 μg/mL and filtered through a 0.45 µm nylon membrane (Merck Millipore (UK) Ltd., Feltham, UK) before being loaded into the system. The sample was analyzed by using Agilent 6530 Accurate-Mass Q-TOF liquid chromatography-mass spectrometry (LC-MS) system with Agilent Zorbax Eclipse XDB-C18 column (2.1 × 50 mm, 1.8 micron) (Agilent Technologies Inc., Mississauga, ON, Canada), and eluted with acetonitrile (Merck) and water using gradient system. The mobile phase started with 99.5% of water and decreased to 50% of water over 10 min, followed by 50% of water and decreased to 0% over 25 min and then held at 0% of water for 5 min, and finally increased to 99.5% of water over 3 min, at a flow rate of 0.25 mL/min. The experiments were performed in the positive ion mode. The flow rate of the drying gas was set at 8 L/min at the temperature of 350 °C. The nebulizer pressure was set at 35 PSIG with the capillary and injection volume set at 3000 V and 5 µL, respectively.

Data of retention time and accurate mass of molecular ions were processed by using qualitative analysis software of Mass Hunter Acquisition Data (Agilent Technologies Inc.) to provide a list of possible molecular formula. Then, accurate mass data and molecular formula were used to corroborate with the data in Dictionary of Natural Products [59] and Dictionary of Marine Natural Products [60]. During the comparison of data, factors like biological resource, compound category and polarity’s reasonability were taken into account to rule out the unreasonable hits [61].

### 3.9. Data Analysis

Mortality of the eggs or larvae or adults in negative control which was 5% to 20% was corrected by Abbott’s formula [62]. The effect of different treatments were compared through one way analysis of variance (ANOVA) followed by Tukey test, using IBM SPSS Statistics version 20 (IBM Corp.). *p* < 0.05 was considered to be statistically significant. 

## 4. Conclusions

The understanding of mosquitocidal potential of *B. pennata* helps in discovering the potential of this natural derived insecticide in the vector control research. This study demonstrated that chloroform extract of *B. pennata* had strong larvicidal, ovicidal as well as oviposition repellence properties against *Ae. aegypti* and *Ae. albopictus* with mild toxic effect against non-target organism (nauplii of *A. salina*). The results from the LC-MS profiling suggested that the contributing compound for the larvicidal effect is an aliphatic compound. Further confirmation of the responsible compound by using other techniques is highly recommended. Since *B. pennata* is a common seaweed found along the coasts of tropical regions, its availability makes further investigation, development and commercialization possible.

## Notes

*Sample Availability:* Samples of the extracts are available from the authors.
